# Atrial fibrillation after transhiatal esophagectomy with transcervical endoscopic esophageal mobilization: one institution’s experience

**DOI:** 10.1186/s13019-018-0746-1

**Published:** 2018-06-19

**Authors:** Elizabeth M. Colwell, Carlos O. Encarnacion, Lisa E. Rein, Aniko Szabo, George Haasler, Mario Gasparri, William Tisol, David Johnstone

**Affiliations:** 1 Cardiothoracic Surgery, Stanford University, 300 Pasteur Dr. Falk Cardiovascular Research Bldg, Stanford, CA 94305-5407 USA; 2 University of Maryland, Division of Cardiac Surgery, 110 S. Paca St. 7th floor, Baltimore, MD 21201 USA; 3 Medical College of Wisconsin, 8701 Watertown Plank Road, PO Box 26509, Milwaukee, WI 53226 USA; 4 Division of Biostatistics, Institute for Health & Equity, Medical College of Wisconsin, 8701 W. Watertown Plank Road, Milwaukee, WI 53226 USA; 5 Division of Cardiothoracic Surgery, HUB for Collaborative Medicine, Medical College of Wisconsin, 8701 Watertown Plank Road, Milwaukee, WI 53226 USA; 6 Division of Cardiovascular and Thoracic Surgery, SSM Heath – St. Mary’s Madison, Madison, WI 53715 USA; 7 Aurora Medical Group CVTS, 2901 W Kinnickinnic River Pkwy Suite 501, Milwaukee, WI 53125 USA

**Keywords:** Esophagectomy, Atrial Fibrillation, Amiodarone

## Abstract

**Background:**

There have been numerous studies regarding atrial fibrillation (AF) associated with cardiac and pulmonary surgery; however, studies looking at esophagectomy and atrial fibrillation are sparse. The goal of this study was to review our institution’s atrial fibrillation rate following esophagectomy in order to better define the incidence and predisposing factors in this patient population.

**Methods:**

A retrospective chart review of all patients undergoing esophagectomy with transcervical endoscopic mobilization of the esophagus (TEEM) at the Medical College of Wisconsin and Affiliated Hospitals from July 2009 through December 2012.

**Results:**

Seventy-one patients underwent TEEM esophagectomy during the study period. Of those, 23 (32.4%) patients developed new atrial fibrillation postoperatively. ICU (Intensive Care Unit) length of stay was 7.1 days for those that did not receive amiodarone, compared to 5.3 days for those that did receive amiodarone (*p* < 0.025). Those that went into AF spent on average 9.3 days in the ICU compared to 4.7 days for their counterparts that did not go into AF (*p* < 0.006). Total length of stay was not statistically different between populations [15.1 +/− 11.3 days compared to 13.5 +/− 9.4 days for those who did not go into AF (*p* < 0.281)]. Receiving preoperative amiodarone was found to reduce the overall incidence of AF. There was a trend towards decreased risk of going into AF in those who received preoperative amiodarone with an adjusted hazard ratio of 0.555 (*p* = 0.057).

**Conclusion:**

Similar to data reported in previous literature, postoperative atrial fibrillation was found to increase ICU length of stay as well as overall length of hospital stay. Preoperative amiodarone administration displayed a trend toward decreasing the rates of atrial fibrillation in patients undergoing TEEM.

## Background

Atrial fibrillation (AF) after thoracic surgery is a common event. Its incidence, risk factors, and consequences have been extensively studied in patients undergoing cardiac surgery. More recently many authors have looked at AF after noncardiac thoracic surgery, reporting its occurrence between 12 and 44% [1] as well as showing an increase in morbidity and mortality in patients developing AF [2–4]. The few reports on postoperative AF after esophageal surgery have found that AF is common. It has been reported in up to 45% of patients following esophagectomy [5]. In 2003 Murthy et al. found AF in 22% of their esophagectomy patients and found it to be a marker for postoperative morbidity and mortality. They reported increased rates of pulmonary complications, anastomotic leak rates, as well as sepsis in patients who had AF [6].

Our study looks at the incidence and treatment of atrial fibrillation in our institution since the implementation of a novel transhiatal esophagectomy technique with endoscopic mobilization of the esophagus (TEEM) and the routine use of perioperative amiodarone.

## Methods

### Patients

A retrospective chart review of all patients undergoing TEEM esophagectomy at the Medical College of Wisconsin and Affiliated Hospitals from July 2009 through December 2012 was performed. This included 80 patients who had an operation performed at Froedtert Hospital or Zablocki VA Hospital in Milwaukee, Wisconsin. Seventy-one patients were included in the final data as one patient from the VA Hospital was excluded due to being unable to obtain a thorough chart review and 8 patients that had pre-operative atrial fibrillation were also excluded from the cohort. Our cohort consisted of a variety of diagnoses such as adenocarcinoma (*n* = 65), squamous cell carcinoma (*n* = 9), achalasia (*n* = 3), GE junction disruption (*n* = 1) and enterocaval fistula (n = 1).

### Data collection

Recorded data included patient demographics, length of stay, length of ICU stay, diagnosis requiring surgery, clinical as well as pathological stage of cancer, medical comorbidities, preoperative chemoradiation status, lymph node dissection, atrial fibrillation during hospital stay, prophylactic amiodarone status, length of surgery, and estimated blood loss. Chart review included extensive review of patient records in EPIC and CPRS (electronic medical records used at our institutions) including preoperative notes, operative records, physician and support staff progress notes, pathology reports and medication administration records. Data collection was approved for use in research by the institutional review board both at Froedtert Hospital and Zablocki VA Hospital.

### Technique

The only surgical approach used in our cohort was a transhiatal esophagectomy with transcervical endoscopic mobilization of the esophagus. Once the abdomen is opened, inspected, and found to be free of any metastatic disease, the left neck is opened in standard fashion medial to the carotid sheath, and the esophagus is encircled with a penrose drain. An endoscopic vein harvest scope is then passed in a posterior plane in order to mobilize the posterior aspect of the esophagus from the thoracic inlet down to the diaphragmatic hiatus. This process is repeated in the anterior and lateral planes as well. Lymph nodes encountered during the dissection are biopsied and sent as specimens to pathology. Tissue division is done with either a Ligasure or Harmonic device (the latter used exclusively in the later portion of this cohort). The esophagus is then divided in the neck. Concurrently, the stomach is mobilized and tubularized in a standard fashion for a transhiatal esophagectomy. The conduit is then passed through the posterior mediastinum and an esophagastric anastomosis is made at the cervical incision, with either a stapled or handsewn anastomosis, or combination of both, depending on patient details and surgeon preference.

Our institution’s therapy of choice for new onset atrial fibrillation for the last several years has been amiodarone. Typically, we have treated our patients who go into stable atrial fibrillation with a 150 mg bolus of amiodarone followed by initiation of an amiodarone drip starting at 1 mg/min for 6 h followed by 0.5 mg/min for 18 h. They are then typically transitioned to j-tube amiodarone as soon as deemed appropriate.

However, starting in January 2011 at Froedtert Hospital we began routinely starting amiodarone the evening before surgery. Patients were admitted for initiation of an amiodarone drip starting at 0.5 mg/min as well as a bowel prep. This amiodarone drip was continued through surgery and transitioned to j-tube dosing once appropriate post-operatively. However, at our other institution, the Zablocki VA Hospital patients did not get admitted the evening before and thus did not get prophylactic amiodarone administration. At this institution, they would only receive amiodarone if they experienced post-operatively AF. All of our patients, at both institutions, generally remained on telemetry throughout their hospital stay.

### Statistical analysis

Statistical analysis was performed by the Biostatistics Division at the Medical College of Wisconsin. The dataset includes 71 patients without pre-op atrial fibrillation, who underwent TEEM esophagectomy from 2009 to 2012. All outcomes (except 30-day survival) were measured during the post-op hospitalization (length of stay varies). All patients except 1 were discharged alive. All patients have adequate follow-up to assess 30-day survival; the minimum follow-up among surviving patients was 33 days.

Predictors and outcomes were compared by pre-op amiodarone status using Wilcoxon rank-sum tests for continuous variables and Fisher’s exact tests for categorical variables.

Predictors and outcomes were compared by post-op day 3 atrial fibrillation status using Wilcoxon rank-sum tests for continuous variables and Fisher’s exact tests for categorical variables. Post-op day 3 status was used because all patients were monitored for atrial fibrillation in hospital at least 3 days (there is no censoring at day 3).

Cox proportional hazards regression analysis (unadjusted and adjusted) was performed for the primary outcome, post-op atrial fibrillation. The cause-specific hazard of atrial fibrillation was modeled using Cox proportional hazards regression. Patients were censored at discharge/death. *P*-values less than 5% were considered significant.

## Results

Seventy-one patients whose ages ranged from 31 to 89 years (mean 61.5 +/− 10.7) were included in the study. There were 63 men and 8 women.

Twenty-eight patients (39.4%) went into atrial fibrillation postoperatively. Sixty-eight percent of our patients underwent preoperative radiation. Of those who had postoperative AF, 67.6% had undergone preoperative radiation; and thus preoperative radiation was not found to be a significant risk factor for atrial fibrillation.

Those who went into AF had a mean age of 66.2 compared with 59.3 in those who did not (*p*-value of < 0.004). The average BMI of our patients was 27.8. We found no difference in the BMI of patients who went into atrial fibrillation and those that did not.

During post-operative follow up, none of the patients who had AF during their hospital stay had AF at their post-operative visit.

Those who went into atrial fibrillation were found to spend 9.3 [1.0–9.0] days in the ICU, compared to 4.7 [0.0–27.0] days in the ICU for those who did not go into AF (*p* < 0.006), however, their overall length of stay was not significantly different, 15.1 [8.0–33.0] days vs. 13.9 [3.0–65.0] days.

Thirty-five (49.3%) of our patients received preoperative amiodarone. Receiving preoperative amiodarone was found to reduce the overall incidence of AF. There was a trend towards decreased risk of going into AF in those who received preoperative amiodarone with an adjusted hazard ratio of 0.555 [95% CI 0.302–1.018 (*p* = 0.057)].

## Discussion

Risk factors for atrial fibrillation following thoracic surgery have spurred much discussion in the literature. Studies have shown male sex, older age, pneumonectomy, esophagectomy, history of smoking, history of congestive heart failure, preoperative supraventricular arrhythmias, and postoperative increase in white blood cell count as risk factors for postoperative arrhythmias [7, 8]. A study by Hahm and colleagues in 2007, looking specifically at esophagectomy patients, found cardiac disease, poor PFTs, cervical anastomosis, elevated CVP, and higher ephedrine doses to be predictors for the occurrence of arrhythmia during surgery [9]. Pneumonia, pleural effusions and elevated c-reactive protein post operative have been associated with increased risk of atrial fibrillation [10].

We found the incidence of new onset postoperative atrial fibrillation in our patient population (all patients undergoing the TEEM approach) to be 39.4%, which is within the wide range reported by other studies [5, 6, 11].

In our group of patient’s arrhythmia most commonly presented on postoperative day 2 or 3. This is consistent with arrhythmia onset after other types of thoracic surgery [2, 8]. Although the reason for a delayed presentation is likely multifactorial, some have speculated that the onset of the arrhythmia is due in part to the resolution of an inflammatory response following surgical trauma which alters the ability of the atrial myocardial cells to respond to catecholamine [11].

Thirty-five of our patients (49.2%) received preoperative amiodarone in attempts to decrease the postoperative AF rate. The use of prophylactic antiarrhythmic medications in thoracic surgery has been highly documented in both cardiac as well as pulmonary surgery. The efficacy of amiodarone for atrial fibrillation prophylaxis has been widely documented in cardiac surgery [12–14]. Guarnieri and colleagues published a trial showing the safe and effective use the use of IV amiodarone in patients undergoing open heart surgery. They found a significant (*p* = 0.01) decrease in the occurrence of postoperative AF in these patients: 35% in patients on amiodarone and 47% in the placebo group. Other groups have found oral amiodarone to be effective as well [12, 14]. Its use has also been documented in lung surgery. Lanza and colleagues showed a decrease in AF rates in patients receiving prophylactic low dose oral amiodarone following pulmonary resections. They found 33% of patients without prophylaxis and 9.7% with prophylaxis (*p* = 0.0253) to have postoperative atrial fibrillation [15]. Even those in whom prophylaxis failed still appeared to benefit as the duration of AF and length of hospital stay were shortened in this group [15]. Although many are concerned about the side effects of amiodarone use, Lanza found no significant complications from prophylactic use in their group of patients.

Tisdale and colleagues published a study in 2010 looking at the efficacy and safety of amiodarone for prevention of atrial fibrillation after transthoracic esophagectomy. They found the incidence of atrial fibrillation requiring treatment was lower in the amiodarone group than in the control group; however there were no significant differences between the groups in terms of overall or ICU length of stay [16]. Our data agrees with Tisdale in that the rate of atrial fibrillation does appear to decrease with prophylactic use of amiodarone. However, our study also suggests that decreasing the rate of AF will decrease the length of stay in the ICU as well as the overall hospital stay. More recently one paper addressed atrial fibrillations affect on long term mortality. Chin et al. showed a significantly lower survival rate in the atrial fibrillation group compared to those who did not have atrial fibrillation *p* = 0.045 via Kaplan Meier survival analysis [17].

There are limitations to this study. The fact that it is a retrospective study brings inherent biases into the study. Being a tertiary care center brings with it a probable patient selection bias as many patients referred to our center are more ill than those receiving care from non-tertiary care centers. In addition, our study size is relatively small as we had only 71 patients undergoing the TEEM approach at the time of data analysis. It may prove beneficial to look at the data again in the near future as many more patients could now be included.

## Conclusion

It is well established that postoperative AF is common in noncardiac thoracic surgery, and it has also been shown to be associated with increased morbidity and mortality as well as increased costs. In our study this is shown by the increased ICU length of stay for patients who go into atrial fibrillation. We found prophylactic amiodarone trended towards a reduction in rates of atrial fibrillation in our patient population, although the rates still remain high. It will be important to continue to investigate ways to recognize those at risk for atrial fibrillation as well as discover ways to continue to decrease the incidence of this arrhythmia in the postoperative period.

## Abbreviations

AFA: Trial fibrillation
TEEM: Transcervical endoscopic mobilization of the esophagus
ICU: Intensive Care Unit
